# Venlafaxine as Monotherapy and in Combination Regimens in Acute Rodent Nociception Experimental Models: A Review

**DOI:** 10.3390/ijms27093944

**Published:** 2026-04-28

**Authors:** Cristina Lungu, Ruxandra-Cristina Marin, Mihnea Costescu, Aurelian Zugravu, Horia Paunescu, Cristina Isabel Ghita, Oana Andreia Coman

**Affiliations:** 1 Department No. 14 of Orthopedics, Anesthesia, and Intensive Care, Faculty of Medicine, “Carol Davila” University of Medicine and Pharmacy, 050098 Bucharest, Romania; cristina.lungu@umfcd.ro; 2 Department of Pharmacology, Clinical Pharmacology and Pharmacotherapy, Faculty of Medicine, “Carol Davila” University of Medicine and Pharmacy, 050474 Bucharest, Romania; aurelian.zugravu@umfcd.ro (A.Z.); horia.paunescu@umfcd.ro (H.P.); isabel.ghita@umfcd.ro (C.I.G.); oana.coman@umfcd.ro (O.A.C.)

**Keywords:** venlafaxine, acute pain, nociception, hot-plate, tail-flick, writhing, opioid potentiation, SNRI, rodent, antinociception

## Abstract

Venlafaxine, a serotonin–norepinephrine reuptake inhibitor, shows analgesic effects in rodents, but its efficacy and pharmacological profile in acute stimulus-evoked nociception may depend on the nociceptive test used and the pharmacological context. The aim of this review was to identify the receptors implicated in venlafaxine antinociceptive effects and to examine which molecular processes most consistently explain its acute antinociceptive profile. We reviewed in vivo rodent studies testing venlafaxine in acute nociceptive assays (writhing, tail-flick, hot-plate, and other eligible acute tests) as monotherapy or associated with other pharmacologically active substances. PubMed/MEDLINE and Web of Science were searched from 1993 to 5 January 2026, and reference lists were also screened. Outcomes were synthesized and stratified by type of nociceptive test and interaction class. Fourteen studies were identified as relevant to the scope of this review. Venlafaxine produced dose-dependent antinociception across tests, reducing writhing and increasing thermal withdrawal latency. Central administration generally yielded effects at lower absolute doses than systemic routes. Interaction studies most consistently supported modulation of opioid receptors (e.g., leftward opioid dose–response shifts and attenuation of morphine tolerance in repeated-exposure designs), with convergent evidence implicating opioid and α_2_-adrenergic mechanisms and context-dependent serotonergic contributions. Additional pathways were variably implicated, including nitric oxide—cyclic guanosine monophosphate (NO–cGMP) signaling and oxidative/mitochondrial processes in opioid tolerance paradigms. Preclinical evidence supports venlafaxine as a modulator of acute nociceptive control with notable opioid-interaction potential. Standardized pharmacodynamic reporting and translationally oriented studies are needed.

## 1. Introduction

Acute pain represents a major reason for emergency presentation, perioperative morbidity, and short-term disability, yet current analgesic strategies are limited by incomplete efficacy and adverse effect profiles. Opioids remain among the most effective pharmacological interventions for acute pain but are limited by dose-dependent respiratory depression, gastrointestinal adverse effects, misuse liability, and the rapid development of tolerance with repeated exposure. These limitations have intensified interest in non-opioid or opioid-sparing strategies that modulate nociceptive processing through different neurochemical mechanisms [1,2].

Pain perception reflects a dynamic interaction between ascending nociceptive signaling and descending modulatory control. Descending inhibitory pathways originating in supraspinal regions, including the periaqueductal gray, rostroventromedial medulla, and locus coeruleus, exert powerful regulatory effects on spinal nociceptive transmission [3]. These circuits depend heavily on serotonergic and noradrenergic neurotransmission to suppress dorsal horn excitability and regulate behavioral pain responses. Pharmacological potentiation of these descending systems therefore represents a central mechanism of endogenous and drug-induced analgesia [4,5].

Within this neurobiological framework, pharmacological modulation of monoaminergic signaling has emerged as a major therapeutic strategy for enhancing endogenous inhibitory pain control. Antidepressants have consistently been recognized to produce clinically analgesic effects that may occur independently of their mood-modulating properties [6,7].

Converging evidence also indicates that disruption of monoaminergic and oxidative neuroregulatory systems through environmental toxic exposure can produce measurable affective and neurobehavioral disturbances, including depressive clinical manifestations observed in populations with chronic aluminum exposure, underscoring the functional sensitivity of these pathways to biochemical modulation [8,9].

Serotonin–norepinephrine reuptake inhibitors (SNRIs) are widely used in chronic pain conditions such as neuropathic pain and fibromyalgia, where enhancement of monoaminergic signaling and descending inhibitory control reduce central sensitization and nociceptive amplification [10,11,12].

Among this class, venlafaxine has been studied due to its well-characterized pharmacological profile and clinical use. Venlafaxine is an SNRI antidepressant introduced in 1993 and widely used in the treatment of major depressive disorder, as well as certain anxiety disorders, depending on regulatory approval. In adult antidepressant therapy, extended-release venlafaxine is typically initiated at 75 mg/day, with lower starting doses (e.g., 37.5 mg/day) used for tolerability, and may be titrated up to 225 mg/day in standard clinical practice [13].

**Figure 1:**
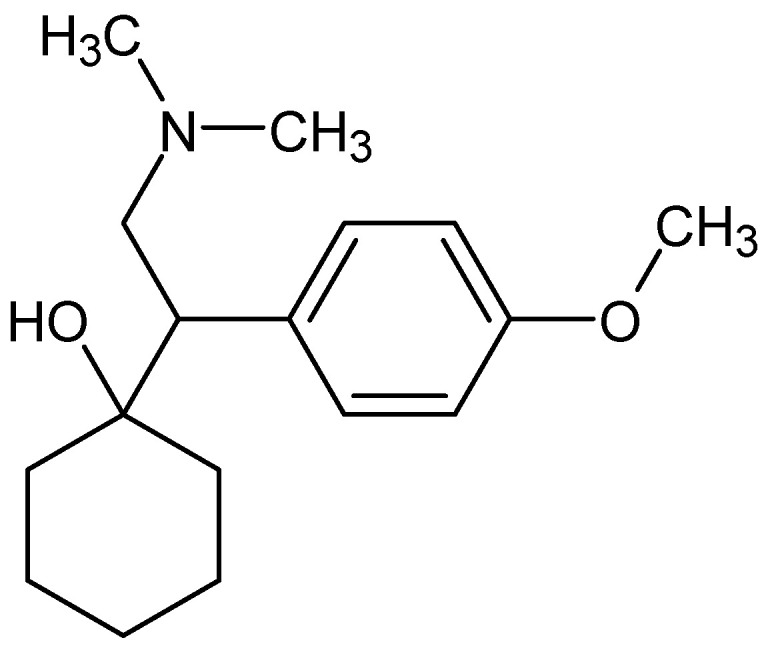
Chemical structure of venlafaxine. Structure was generated using ACD/ChemSketch (version 12.1.0.31258), Advanced Chemistry Development, Inc., Toronto, ON, Canada.

Chemically, venlafaxine is a phenethylamine derivative, most commonly administered as venlafaxine hydrochloride. Its molecular structure (Figure 1) reflects the basis of its pharmacological activity, especially its ability to interact with monoamine transporters through its amine and aromatic functional groups.

Pharmacodynamically, it exhibits dose-dependent inhibition of monoamine transporters, with predominant serotonergic effects at lower doses and increasing noradrenergic contribution at higher doses, a profile that may influence its effects on pain modulation. In addition, venlafaxine is extensively metabolized to its active metabolite, O-desmethylvenlafaxine (ODV), which contributes to its overall pharmacological activity and may affect the duration and consistency of central monoaminergic modulation. These pharmacological and structural characteristics underlie its dual monoaminergic mechanism of action, which is relevant not only for antidepressant effects but also for potential modulation of nociceptive processing [14,15].

Consistent with this mechanism, SNRIs block serotonin and norepinephrine transporters, increasing synaptic monoamine availability within pain-modulatory circuits and strengthening endogenous inhibitory tone [3]. However, extrapolation of these clinical observations to acute nociception remains uncertain. Acute pain is largely driven by rapid stimulus-evoked activation of peripheral and central pathways, whereas chronic pain states involve sustained neuroplasticity and central sensitization. These biological differences complicate direct translation of monoaminergic analgesic mechanisms across pain states [12,16]. Nevertheless, given its pharmacological profile, venlafaxine has been increasingly investigated in experimental models of acute nociception as a potential modulator of descending inhibitory control.

To investigate these pharmacological effects under controlled conditions, experimental models of acute nociception in rodents are commonly employed. Acute nociceptive assays in rodents involve partially distinct anatomical and physiological substrates: the writhing test predominantly reflects chemically induced visceral nociception, the tail-flick test is often considered a spinally mediated reflex dominated by segmental processing, and the hot-plate test requires supraspinal integration and is particularly sensitive to centrally acting analgesics and drug–drug interactions [17].

However, interpretation of findings across these models is complicated by substantial heterogeneity in experimental design. Studies differ in species, dosing regimens, administration routes, pharmacological probes, and the magnitude of observed effects varies across nociceptive tests [18,19]. In addition, the reporting of pharmacodynamic outcomes is not uniform across studies. Quantitative interaction outcomes such as receptor antagonism profiles, dose–response relationships, and tolerance modulation are unevenly distributed across experimental designs, resulting in fragmented and sometimes inconsistent findings.

To our knowledge, no review has comprehensively synthesized acute nociceptive outcomes stratified simultaneously by nociceptive assay level and pharmacological interaction class. Such stratification is necessary to determine whether venlafaxine’s antinociceptive profile reflects generalized monoaminergic modulation or assay-specific recruitment of distinct inhibitory mechanisms. Addressing this gap, the aim of the present review is to evaluate the antinociceptive effects of venlafaxine in acute preclinical pain models.

The primary objective is to assess venlafaxine’s antinociceptive efficacy across distinct levels of nociceptive processing. Secondary objectives are to characterize pharmacological interactions, evaluate dose–response relationships, identify receptor and signaling mechanisms underlying observed effects, and compare outcomes across peripheral, spinal, and supraspinal levels. By integrating antinociceptive outcomes with mapping of opioid, adrenergic, serotonergic, and intracellular signaling pathways, this review aims to clarify at which nervous level venlafaxine produces antinociception, and which molecular processes most consistently explain its acute antinociceptive profile.

## 2. Literature Identification Approach

To support this review, the literature on venlafaxine in acute rodent nociception models was examined in PubMed/MEDLINE and Web of Science from 1993, the year in which venlafaxine was introduced by the U.S. Food and Drug Administration (FDA), to 5 January 2026. Search terms combined venlafaxine-related keywords with descriptors of experimental nociception and acute pain assays, including hot-plate, tail-flick, writhing test, acetic acid, opioid interaction, and animal model. Additional searches included terms related to pharmacological interactions: morphine, naloxone, clonidine, yohimbine, serotonergic antagonists, and nitric oxide. Reference lists of relevant articles were also screened to identify additional studies.

The review focused on original in vivo rodent studies evaluating venlafaxine in acute nociceptive tests, either as monotherapy or in combination with other pharmacological agents. Emphasis was placed on studies reporting behavioral nociceptive outcomes and on comparisons across nociceptive paradigms, routes of administration, and pharmacological interaction contexts.

Because the available evidence was heterogeneous in design, species, dosing protocols, and outcome reporting, findings were synthesized narratively and organized according to nociceptive assay type and pharmacological context.

## 3. Relevant Sections

### 3.1. Characteristics of Included Studies

The studies discussed in this review were conducted in mice and rats, with mice representing the majority of experimental models. Sample sizes ranged from 6 to 12 animals per group in most studies, although group size reporting was incomplete in several reports. Sex of experimental animals was reported in nine of 14 studies, with male animals predominating; female inclusion was limited.

Experimental paradigms comprised validated acute nociceptive assays representing different levels of nociceptive processing, including the hot-plate test (acute supraspinal thermal nociception evaluation), tail-flick test (acute spinal thermal nociception evaluation), and acetic-acid–induced writhing test (acute chemically induced visceral nociception model with predominant peripheral and inflammatory mechanisms). Several studies used more than one nociception test within the same experimental protocol. Venlafaxine was administered by multiple routes, including intraperitoneal, subcutaneous, oral, intracerebroventricular, and intrathecal. Dosing regimens varied substantially across studies. Systemic doses most commonly ranged from 5 to 80 mg/kg, while central administration doses ranged from 25 to 100 µg intra-cerebrospinal fluid or intra-nuclei.

Experimental designs included venlafaxine monotherapy and pharmacological interaction tests involving opioid agonists, receptor antagonists, serotonergic ligands, adrenergic modulators, nitric oxide pathway inhibitors, calcium channel blockers, and other pharmacological agents.

### 3.2. Distribution of Experimental Tests and Pharmacological Context

Across the included literature, a supraspinal integrated thermal assay was the most commonly used experimental setting (hot-plate outcomes reported in 10 of 14 studies), and this test was most frequently used for detailed pharmacodynamic characterization. In contrast, spinal reflex (tail-flick; six studies) and visceral assays (writhing; five studies) were less frequently used in interaction-oriented designs but still demonstrated antinociceptive effects across multiple dosing and administration routes.

Ten studies evaluated venlafaxine as monotherapy. Opioid interaction experimental tests were reported in seven studies, and non-opioid pharmacological modulators were examined in nine studies. Five studies implemented more than one nociceptive assay within the same experimental framework.

Five studies evaluated venlafaxine across more than one nociceptive test within the same experimental team. These studies concomitantly used the writhing, tail-flick, and hot-plate tests. Consequently, one can compare results in the spinal and supraspinal thermal tests.

The characteristics and outcomes of venlafaxine as monotherapy studies are summarized in Table 1.

The characteristics and outcomes of opioid interaction studies are summarized in Table 2.

The characteristics and outcomes of non-opioid interaction studies are summarized in Table 3.

## 4. Discussion

The present review identified 14 preclinical studies and indicated that venlafaxine produces measurable antinociceptive effects in acute nociceptive tests. The apparent intensity and interaction profile with other substances depend on the level (supraspinal or spinal and peripheral) of nociceptive processing evaluated by the tests and also on the pharmacological context in which venlafaxine is administered. This pattern aligns with the idea outlined in the Introduction, in which venlafaxine functions less as a uniform “monoaminergic analgesic” and more as a modulator of the inhibitory control of pain stimuli. The evidence is most consistent with the fact that venlafaxine influences nociception through recruitment of descending inhibitory circuits and through receptor-level interactions (particularly with opioid and adrenergic systems) and also by downstream cellular adaptations that become especially visible in repeated-opioid or tolerance-oriented tests.

### 4.1. Antinociceptive Effects of Venlafaxine as Monotherapy

Venlafaxine administered alone produced measurable changes in nociceptive behavior across all three primary assays.

In thermal nociceptive assays, venlafaxine decreased the thermal nociceptive response (e.g., paw licking, jump or tail-flick latency) in nine of 10 studies evaluating hot-plate or tail-flick responses. Reported maximal latency ranged from approximately 15% to 70% above baseline depending on dose and administration route [20,21]. Dose-dependent effects were reported in several studies, and ED_50_ values for hot-plate antinociception were estimated in two independent experiments, both reporting values in the mid-dose systemic range.

In chemical-induced nociception models, venlafaxine reduced abdominal constriction responses in all studies evaluating the writhing test. Reported maximal inhibition ranged from approximately 20% to greater than 60% relative to control conditions, depending on dose and experimental design. One study reported an ED_50_ value of 12.37 mg/kg for writhing inhibition [26,28].

Central administration produced antinociceptive effects at lower doses than systemic administration in studies directly comparing routes of delivery. Antinociceptive effects following intracerebroventricular administration were observed across writhing, tail-flick, and hot-plate tests [27].

### 4.2. Venlafaxine–Opioid Pharmacological Interactions

Seven studies evaluated venlafaxine in combination with opioid agonists and or antagonists.

Across dose–response experiments, co-administration of venlafaxine at an ineffective dose with opioid agonists produced reductions in opioid ED_50_ values in multiple studies [20,21]. Reported left-sided shifts included reductions of approximately two- to six-fold for morphine and other opioid agonists, depending on receptor subtype and experimental conditions.

Opioid receptor antagonists (naloxone, Nor-BNI, naltrindole, β–FNA, naloxonazine) reversed or attenuated venlafaxine-associated antinociceptive effects in several experiments. Reversal was complete in some hot-plate assays and partial in others, depending on antagonist type and dosing [20,21,22,24].

Under conditions of chronic treatment, venlafaxine has been shown to significantly maintain antinociceptive efficacy during prolonged morphine exposure. Specifically, [34] demonstrated that daily co-administration of venlafaxine prevents the development of opioid tolerance by suppressing neuroinflammatory pathways. One study evaluating methadone reported no interaction under the tested conditions [30].

### 4.3. Venlafaxine Interactions with Non-Opioid Modulators

Nine studies examined venlafaxine in combination with non-opioid pharmacological agents.

Adrenergic modulation was examined in multiple experiments. Alpha2-adrenergic receptor agonists enhanced antinociceptive responses in thermal assays, while α_2_ receptor antagonists reduced antinociceptive effects in selected experiments [20,21].

Manipulation of serotonergic receptor activity produced variable nociception outcomes across experimental conditions, including both enhancement and attenuation of antinociceptive responses depending on receptor subtype and assay [23]. Nitric oxide synthase inhibition did not consistently alter venlafaxine-induced responses in acute thermal assays [22,34]. Additional studies reported enhanced antinociceptive responses following co-administration with calcium channel blockers, plant-derived compounds, and catecholaminergic reuptake inhibitors. Synergistic antinociceptive interaction was reported in one study using plant extract [28].

### 4.4. Quantitative Characteristics of Antinociceptive Outcomes

Across all assays, venlafaxine produced measurable antinociceptive effects within dose ranges typically between 5 and 80 mg/kg for systemic administration and 25 to 100 µg for central administration. In the writhing test, dose-dependent inhibition of the nociceptive response was consistently observed starting at 10 mg/kg [28], with other reports requiring up to 25 mg/kg for a highly significant effect [26]. Thermal nociception assays exhibited a similar threshold-dependency; significant increases in latency were noted starting at 10 mg/kg in the hot-plate test [29] and 25 mg/kg in the tail-flick assay [26]. Notably, while these lower doses initiate antinociception, higher doses approaching 40–50 mg/kg are generally required to achieve the ED_50_ in acute thermal models [20,21].

Pharmacological interaction experiments frequently quantified antinociceptive efficacy using dose–response curve shifts. Reported opioid ED_50_ reductions ranged from approximately two-fold to greater than five-fold depending on receptor subtype and experimental conditions.

Studies involving central drug delivery reported percentage changes in nociceptive responses across multiple assays within the same experimental framework.

### 4.5. Hypothesis on Possible Molecular Interactions of Venlafaxine and Desvenlafaxine with Monoaminergic, Opioid and Other Mediators of Pain Circuits

Due to the fact that venlafaxine proved to have an analgesic effect in the hot-plate test, which is a supraspinal integrated thermal assay, one can suppose that venlafaxine’s mechanism of action implies descending monoaminergic inhibitory networks and probably an interaction with the opioid system.

At the molecular level, venlafaxine inhibits serotonin (SERT) and norepinephrine (NET) transporters, increasing synaptic 5-HT and NE in circuits that contribute to descending inhibitory control. These monoamines modulate spinal nociceptive transmission through well-described descending pathways that suppress dorsal horn excitability. However, across the reviewed studies, venlafaxine’s antinociceptive effects were repeatedly sensitive to opioid and adrenergic interference, indicating that monoamine reuptake inhibition alone does not fully account for the observed behavioral outcomes in acute nociceptive tests [3,20].

This assay-level interpretation is consistent with recent models of endogenous pain regulation, which emphasize a modulation within descending pain inhibitory systems. Rather than acting through a single receptor mechanism, centrally acting agents influence nociception by shifting the balance between facilitatory and inhibitory outputs across interconnected brainstem–spinal networks [35,36].

Differences between peripheral visceral analgesia models, spinal reflex assays, and supraspinal integrated tests can be interpreted within the hierarchical organization of nociceptive processing. Visceral analgesic tests, such as the writhing test, largely reflect peripheral nociceptor activation and inflammatory mediator signaling, spinal reflex tests emphasize dorsal horn processing, and hot-plate-type tests depend on supraspinal integration and descending modulation. Given this organization, agents that strengthen descending inhibitory tone would be expected to show effects in centrally mediated behaviors of pain avoidance [37,38].

This interpretation aligns with established models of descending pain modulatory circuitry linking cortical, midbrain, and medullary structures to spinal nociceptive transmission. Venlafaxine’s enhancement of serotonergic and noradrenergic signaling provides multiple opportunities to influence key nodes, including the periaqueductal gray, rostroventromedial medulla, and locus coeruleus, which coordinate inhibitory and facilitatory outputs and contribute to endogenous and opioid-mediated analgesia. Increased monoaminergic tone may therefore lead to inhibition of spinal nociceptive gain and to enhancement of centrally mediated antinociception [4,39].

A defining feature of the reviewed interaction literature (seven studies) is the recurring functional coupling between venlafaxine and opioid signaling. In several dose–response tests, venlafaxine reduced opioid ED_50_ values and thus enhanced opioid analgesic efficacy, while antagonists used in different tests frequently diminished venlafaxine-associated antinociception. These findings suggest that venlafaxine can modify opioid system responsiveness under specific experimental conditions rather than simply adding an independent analgesic effect [20,21].

Noradrenergic mechanisms, particularly α_2_-adrenergic-receptor-mediated inhibition, appear in two studies to contribute to the increase in the descending inhibition at the spinal level and may interact with opioid-mediated supraspinal modulation [20,21]. In contrast, serotonergic interactions are more variable across studies and depend on receptor subtype, anatomical site, and stimulus type. 5-HT_1A_ receptor antagonists, for example, produced enhancement of venlafaxine’s antinociceptive effect in an acute pain model or a decrease in this effect in a tonic pain model [23,32].

**Figure 2:**
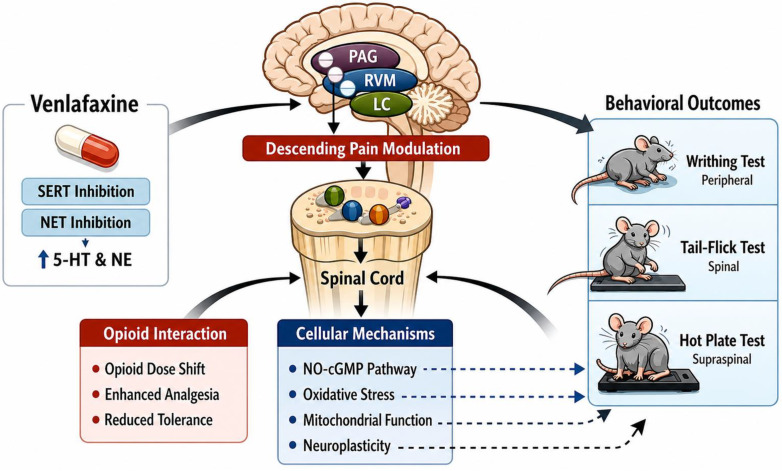
Integrated framework of venlafaxine antinociception across levels of nociceptive processing. Venlafaxine inhibits serotonin (SERT) and norepinephrine (NET) transporters, increasing synaptic 5-HT and NE within descending pain modulatory circuits, including the periaqueductal gray (PAG), rostroventromedial medulla (RVM), and locus coeruleus (LC). Enhanced monoaminergic tone modulates spinal dorsal horn excitability through α_2_-adrenergic and opioid receptor interactions. Preclinical evidence demonstrates modulation of opioid molecular effects, including leftward dose–response shifts, potentiation of opioid analgesia, and attenuation of tolerance development. Additional intracellular mechanisms implicated in experimental paradigms include nitric oxide–cGMP signaling, oxidative stress regulation, mitochondrial function, and adaptive plasticity processes. The antinociception is evidenced across peripheral (writhing), spinal (tail-flick), and supraspinal (hot-plate) nociceptive assays. The figure was created in BioRender. Calin, M. (2026) https://BioRender.com/q3jq4ha (accessed on 19 March 2026).

Based on the integrated interpretation of the available experimental evidence, a framework of venlafaxine antinociception is shown in Figure 2.

An additional pharmacological dimension relevant to venlafaxine antinociception is the activity of its primary active metabolite, ODV. Venlafaxine undergoes extensive hepatic metabolism primarily via murine equivalent of human CYP2D6-mediated O-demethylation, producing ODV, which has serotonin–norepinephrine reuptake inhibitory activity comparable to the parent compound. Both venlafaxine and ODV penetrate the central nervous system and contribute to sustained monoaminergic modulation of pain-regulatory circuits. Pharmacokinetic studies indicate that ODV exhibits a longer elimination half-life than venlafaxine and constitutes a major circulating active metabolite following systemic administration. This profile may help explain the persistence and reproducibility of venlafaxine-induced antinociception across tests requiring prolonged central modulation. Furthermore, interindividual and interspecies variability in CYP2D6-mediated metabolism may represent an underrecognized source of heterogeneity in experimental analgesic outcomes. Consideration of venlafaxine–ODV pharmacokinetic dynamics is therefore relevant when interpreting dose–response relationships and cross-study variability in preclinical tests of antinociceptive effects [40,41].

Beyond monoaminergic reuptake and pharmacokinetic considerations, several studies suggest that venlafaxine can influence intracellular and adaptive signaling pathways that become most evident in opioid tolerance tests. In acute hot-plate assays explicitly testing interaction with opioid and nitric oxide systems, available evidence supports opioid involvement in venlafaxine-associated antinociception, whereas nitric oxide synthase inhibition does not consistently attenuate this effect. In contrast, in tolerance- and dependence-oriented tests, modulation of L-arginine/NO/cGMP signaling has been implicated in venlafaxine-associated attenuation of opioid tolerance and related adaptive processes [22,31,34].

In an opioid tolerance test, venlafaxine has been reported to attenuate the development of morphine tolerance and dependence, implicating L-arginine/NO/cGMP signaling and associated neuroinflammatory or oxidative mechanisms [34].

Emerging data increasingly identify oxidative stress signaling and mitochondrial dysfunction as central regulators of inflammatory amplification and cellular injury in acute pathophysiological conditions, further supporting the biological plausibility of redox-dependent mechanisms contributing to venlafaxine-associated modulation of nociceptive plasticity [42].

More recently, co-administration of venlafaxine with calcium channel blockers was shown to enhance morphine’s acute analgesia and to mitigate morphine-associated mitochondrial oxidative stress during tolerance experiments, further supporting involvement of cellular stress and excitability pathways [29].

In the overall interaction profile across non-opioid studies (nine studies) adrenergic modulation frequently altered venlafaxine-associated effects in thermal assays, while serotonergic receptor manipulations produced conflicting results depending on receptor subtype and experimental design.

Beyond classical neuronal receptor mechanisms, an additional interpretive layer concerns non-neuronal contributors to nociceptive processing. Microglia and astrocytes regulate synaptic transmission and nociceptive sensitivity via cytokines, reactive oxygen species, and neuromodulatory mediators that influence neuronal excitability. Evidence from experimental and translational literature indicates that antidepressants can attenuate microglial activation and modulate neuroinflammatory signaling pathways linked to pain amplification and central sensitization, although these mechanisms are more extensively characterized in neuropathic and chronic pain models than in acute stimulus-evoked tests [43].

Antidepressant-associated modulation of microglial reactivity and inflammatory mediator signaling can alter pain-related behavioral sensitivity in relevant models, reinforcing neuroimmune regulation as a plausible contributor to antidepressant–pain interactions in settings where neuroinflammatory tone is elevated [44].

From a translational perspective, the preclinical interaction profile supports the hypothesis that venlafaxine could function as a centrally acting adjunct capable of modifying opioid responsiveness under certain conditions. Across the included interaction studies, venlafaxine was repeatedly associated with opioid dose–response shifts, antagonist sensitivity, and modulation of behavioral outcomes during repeated opioid exposure, providing experimental evidence of pharmacodynamic interaction in acute nociceptive paradigms. This interpretation is conceptually aligned with multimodal analgesia assays in which agents targeting convergent mechanisms are combined to enhance analgesia while limiting opioid exposure. However, the present evidence base is preclinical, assay-dependent, and heterogeneous in design, limiting direct extrapolation to clinical acute pain settings [45,46].

However, extrapolation from preclinical findings to clinical practice requires caution. Commonly used stimulus-evoked nociceptive assays in rodents capture only selected components of pain and do not fully represent the multidimensional human pain experience. In particular, neuroplastic mechanisms such as central sensitization, which are critical drivers of pain amplification, are seldom represented across acute nociceptive assays [12,16], further limiting translation of experimental interaction effects to human acute pain conditions [37,47].

### 4.6. Reporting Quality and Methodological Considerations

Assessment of reporting quality across the reviewed literature identified variability in the description of experimental design and bias-reducing procedures [48].

Random allocation of animals to experimental groups was explicitly reported in five of 14 studies. Blinded outcome assessment was reported in three studies. Allocation concealment was not reported in any study. Sample size calculation was not reported in any study.

Baseline group comparability was described in six studies. Attrition handling was reported in four studies. Housing and husbandry conditions were incompletely reported in several experiments.

Descriptions of drug administration and experimental testing procedures were generally detailed, but reporting of methodological safeguards to reduce experimental bias was inconsistent across the literature.

Given the diversity of experimental designs, species, dosing protocols, administration routes, and outcome reporting formats, a quantitative pooled analysis was not considered appropriate. Instead, findings were synthesized using structured comparison across nociceptive paradigms and pharmacological contexts. Attention was placed on the reproducibility of venlafaxine antinociception, the magnitude and direction of interaction effects, and the consistency of receptor-level mechanistic findings.

One important bias of the studies included in this review is the fact that in many studies, rats were used, a fact that can lead to misinterpretation of their results, due to the fact that these analgesic tests are mainly described and standardized in mice.

An additional methodological consideration relevant to the interpretation of antinociceptive outcomes is the potential influence of antidepressants on motor activity and muscle tone. Antidepressant compounds, including venlafaxine, may alter locomotor activity, arousal state, or neuromuscular function, which could affect behavioral responses in nociceptive assays. Although commonly used tests such as the hot-plate, tail-flick, and writhing paradigms are designed to reflect nociceptive processing, they rely on motor outputs that may be partially influenced by non-nociceptive factors. Therefore, changes in response latency or frequency should be interpreted with caution, particularly at higher doses, where sedative or stimulatory effects may occur. This limitation is inherent to behavioral nociceptive testing and should be considered when comparing results across studies [49,50].

In addition, alternative behavioral approaches such as mechanical sensitivity testing using von Frey filaments provide a minimally aversive method for assessing nociceptive thresholds in rodents. These tests are widely used to evaluate mechanical nociception and offer advantages in terms of reduced stress and improved reproducibility compared to more aversive stimulus-evoked assays. Although von Frey paradigms are more commonly applied in models of inflammatory or neuropathic pain, their inclusion in experimental designs may enhance the methodological robustness and translational relevance of preclinical nociception studies [51,52].

### 4.7. Integrated Summary of Experimental Findings

Across the included studies, venlafaxine administration produced measurable changes in nociceptive behavior across peripheral chemical, inflammatory (formalin test), spinal reflex, and supraspinal integrated tests. Significant effects were reported in all major assays examined.

Pharmacological interaction experiments demonstrated modifications of behavioral responses to pain under combined drug administration conditions, including dose–response shifts and increased sensitivity of µ-, δ-, $k_{1}$-, and $k_{2}$-opioid receptor subtypes, as well as $\alpha_{2}$-adrenergic receptors. Furthermore, these interactions lead to altered responses during repeated opioid exposure, specifically by attenuating the development of analgesic tolerance and maintaining receptor responsiveness [20,21].

Considerable variability was observed across species, dosing regimens, routes of administration, and outcome reporting formats. The evaluation of biological sex remains important, given established sex differences in nociceptive processing and the persistent underrepresentation of female animals in preclinical analgesic research. This variability limited direct quantitative comparison across studies, supporting the present narrative synthesis organized by nociceptive test and pharmacological context.

## 5. Conclusions

Venlafaxine produces reproducible antinociceptive effects across peripheral chemical, spinal reflex and supraspinal thermal stimuli avoidance in rodent acute pain models. The available scientific evidence supports venlafaxine as a modulator of nociceptive processing involving concomitant monoaminergic (SNRI), opioid, and α_2_-adrenergic mechanisms rather than monoamine reuptake inhibition alone.

Across pharmacological interaction studies, venlafaxine consistently modifies opioid responsiveness, including dose–response shifts and altered behavioral outcomes during repeated opioid exposure. These findings provide a biologically plausible rationale for considering venlafaxine within potentially opioid-sparing analgesic strategies, although current evidence is limited to a small number of experimental pain settings. Substantial methodological heterogeneity and the restricted scope of acute nociceptive assays limit direct clinical extrapolation.

## 6. Future Directions

Future research should prioritize methodological standardization, harmonized pharmacodynamic reporting, and integration of pharmacokinetic–pharmacodynamic modeling to improve translational interpretation. Additional studies integrating behavioral, molecular, and pain circuit-level approaches are needed to clarify the anatomical and mechanistic specificity of venlafaxine-associated antinociception. Controlled translational studies, and ultimately clinical trials evaluating venlafaxine as an adjunct to standard analgesic regimens, will be necessary to determine whether these preclinical interaction patterns translate into meaningful benefit in human acute pain management.

## Figures and Tables

**Table 1:** Antinociceptive effects of venlafaxine as monotherapy in acute nociceptive rodent models.

Study	Species/Model	Route of Administration	Venlafaxine Dose	Test	Outcomes	Experimental Objective of the Study
Schreiber et al., 1999 [20]	Male ICR mice	i.p.	5–30 mg/kg	Hot-plate	Dose-dependent latency increase; ED_50_ = 46.7 mg/kg	D-E R for venlafaxine in hot-plate test.
Schreiber et al., 2002 [21]	Male ICR mice	i.p.	1–30 mg/kg	Hot-plate	Dose-dependent latency increase; ED_50_ = 46.7 mg/kg	D-E R for venlafaxine in hot-plate test.
Gultekin & Ahmedov, 2006 [22]	Wistar rats	i.p.	25 mg/kg	Hot-plate	Significant latency increase (*p* < 0.05)	D-E R for venlafaxine in hot-plate test.
Berrocoso & Mico, 2009 [23]	CD1 mice	i.p.	20–80 mg/kg	Hot-plate	Dose-related latency increase; significant at 80 mg/kg	D-E R for venlafaxine in hot-plate test.
Sikka et al., 2011 [24]	Albino mice	s.c.	40–50 mg/kg	Tail-flick	Significant latency increase	The antinociceptive efficacy of venlafaxine in tail -flick test.
Ozdemir et al., 2012 [25]	Wistar rats	s.c.	20 mg/kg	Tail-flick	Significant latency increase (*p* < 0.01)	The antinociceptive efficacy of venlafaxine in tail -flick test.
				Hot-plate	Significant latency increase (*p* < 0.01)	The antinociceptive effect of venlafaxine in hot-plate test.
Kothari et al., 2013 [26]	Albino mice	p.o.	25 mg/kg	Writhing	Significant inhibition of writhing	To evaluate venlafaxine effects in a visceral chemical pain model.
				Tail-flick	Significant latency increase	The antinociceptive efficacy of venlafaxine in tail -flick test.
Motaghinejad et al., 2014 [27]	Wistar rats	i.c.v.	25–100 µg	Writhing	20–37% inhibition dose-dependent	D-E R for venlafaxine effects in a visceral chemical pain model.
				Tail-flick	23–69% latency increase	D-E R for venlafaxine in tail-flick test.
				Hot-plate	15–29% latency increase	To assess central venlafaxine effects on supraspinal nociceptive processing.
Mansouri et al., 2015 [28]	Swiss mice	i.p.	3–60 mg/kg	Writhing	Dose-dependent inhibition; ED_50_ = 12.37 mg/kg	D-E R for venlafaxine effects in a visceral chemical pain model.
Soleimanii et al., 2024 [29]	NMRI mice	i.p.	10–40 mg/kg	Hot-plate	Dose-dependent latency increase	D-E R for venlafaxine effects in hot-plate test.

D-E R, dose–effect relationship; i.p., intraperitoneal; s.c., subcutaneous; p.o., oral; i.c.v., intracerebroventricular; ED_50_, median effective dose. Thermal latency: time from stimulus onset to the thermal nociceptive response (e.g., paw licking, jump or tail-flick latency). Writhing inhibition: reduction in abdominal constriction frequency relative to control.

**Table 2:** Pharmacodynamic interactions between venlafaxine and opioid agents in acute nociceptive rodent models.

Study	Species/Model	Venlafaxine Dose; Route	Opioid/Antagonist; Route of Administration	Test	Outcomes	Experimental Objective of the Study
Schreiber et al., 1999 [20]	Male ICR mice	2.5 mg/kg; i.p.	Morphine; s.c.	Hot-plate	Morphine ED_50_ reduced 5.8 → 1.8 mg/kg	D-E R for morphine in interaction with ineffective single dose of venlafaxine in hot-plate test.
			DPDPE (δ agonist); i.t.	Hot-plate	ED_50_ reduced 320 → 90 ng	D-E R for DPDPE i.t. in interaction with ineffective single dose of venlafaxine in hot-plate test.
			U50488H (κ agonist); s.c.	Hot-plate	ED_50_ reduced 5.7 → 1.0 mg/kg	D-E R for U50488H in interaction with ineffective single dose of venlafaxine in hot-plate test.
			Nalorphine (µ antagonist and k agonist); s.c.	Hot-plate	ED_50_ reduced 29.3 → 0.4 mg/kg	D-E R for nalorphine in interaction with ineffective single dose of venlafaxine in hot-plate test.
		30 mg/kg; i.p.	Naloxone (non-selective antagonist); s.c.	Hot-plate	Antinociception reversed	D-E R for venlafaxine in interaction with naloxone in hot-plate test.
			Nor-BNI (κ antagonist); s.c.	Hot-plate	Antinociception reversed	D-E R for venlafaxine in interaction with Nor-BNI in hot-plate test.
			Naltrindole (δ antagonist); s.c.	Hot-plate	Antinociception reversed	D-E R for venlafaxine in interaction with naltrindole in hot-plate test.
			β–FNA (µ antagonist); s.c.	Hot-plate	Partially antinociception reversed	D-E R for venlafaxine in interaction with β–FNA in hot-plate test.
			Naloxonazine, (μ_1_ antagonist); s.c.	Hot-plate	Partially antinociception reversed	D-E R for venlafaxine in interaction with naloxonazine in hot-plate test.
Schreiber et al., 2002 [21]	Male ICR mice	2.5 mg/kg; i.p.	Morphine; s.c.	Hot-plate	ED_50_ reduced 5.4 → 2.4 mg/kg	To assess venlafaxine influence on morphine dose–response antinociception.
			DPDPE; i.t.	Hot-plate	ED_50_ reduced 393 → 130 ng	To evaluate venlafaxine interaction with δ-opioid receptor agonism.
			U50488H; s.c.	Hot-plate	ED_50_ reduced 5.5 → 1.6 mg/kg	To evaluate venlafaxine interaction with κ-opioid receptor agonism.
			Nalorphine; s.c.	Hot-plate	ED_50_ reduced 31.1 → 2.7 mg/kg	To examine venlafaxine modulation of mixed opioid receptor activity.
		30 mg/kg; i.p.	Naloxone; s.c.	Hot-plate	Antinociception reversed	To determine opioid receptor contribution to effective analgesic dose venlafaxine antinociception
			Nor-BNI (κ antagonist); s.c.	Hot-plate	Antinociception reversed	D-E R for venlafaxine in interaction with Nor-BNI in hot-plate test.
			Naltrindole (δ antagonist); s.c.	Hot-plate	Antinociception reversed	D-E R for venlafaxine in interaction with naltrindole in hot-plate test.
			β–FNA (µ antagonist); s.c.	Hot-plate	Partially antinociception reversed	D-E R for venlafaxine in interaction with β–FNA in hot-plate test.
			Naloxonazine, (μ_1_ antagonist); s.c.	Hot-plate	Partially antinociception reversed	D-E R for venlafaxine in interaction with naloxonazine in hot-plate test.
Gultekin & Ahmedov, 2006 [22]	Wistar rats	25 mg/kg; i.p.	Naloxone; i.p.	Hot-plate	Partially antinociception reversed	To assess the role of opioid receptors in venlafaxine-induced antinociception.
Sikka et al., 2011 [24]	Albino mice	30 mg/kg, s.c.	Morphine; s.c.	Tail-flick	Increased latency of the nociceptive response to thermal stimulus	To evaluate combined effects of venlafaxine and morphine in spinal nociception.
		40–50 mg/kg; s.c.	Naloxone; s.c.	Tail-flick	No increase in latency of the nociceptive response to thermal stimulus	To determine the effect of opioid receptor blockade on venlafaxine antinociception.
Ozdemir et al., 2012 [25]	Wistar rats	20 mg/kg; s.c.	Morphine; s.c.	Tail-flick	Increased MPE	To examine venlafaxine effects on morphine analgesia in acute nociception.
				Hot-plate	Increased MPE	To examine venlafaxine effects on morphine analgesia in supraspinal nociception.
Schreiber et al., 2014 [30]	Male ICR mice	2.5 mg/kg; i.p.	Methadone; s.c.	Hot-plate	No interaction in antinociceptive effect	To evaluate interaction between venlafaxine and methadone in acute nociception.
Mansouri et al., 2018 [31]	Swiss mice	5–40 mg/kg; i.p.	Repeated morphine; s.c.	Hot-plate	Prevention of tolerance	To investigate the effect of venlafaxine on development of morphine tolerance during repeated administration.

D-E R, dose–effect relationship, i.p., intraperitoneal; s.c., subcutaneous; i.t., intrathecal; ED_50_, median effective dose; MPE, maximal possible effect. Thermal latency: time from stimulus onset to the thermal nociceptive response (e.g., paw licking, jump or tail-flick latency). Writhing inhibition: reduction in abdominal constriction frequency relative to control.

**Table 3:** Pharmacological interactions between venlafaxine and non-opioid modulators of nociceptive processing in acute nociceptive rodent models.

Study	Species/Model	Venlafaxine; Dose; Route	Non-opioid Modulator; Route of Administration	Test	Outcomes	Experimental Objective of the Study
Schreiber et al., 1999 [20]	Male ICR mice	30 mg/kg; i.p.	Clonidine (α_2_ agonist); s.c.	Hot-plate	6-fold ED_50_ reduction of clonidine 0.34 → 0.06 mg/kg	To evaluate interaction between venlafaxine and α_2_-adrenergic receptor activation.
			Phentolamine; s.c.	Hot-plate	No significant changes	To assess the effect of non-selective α-adrenergic receptor blockade on venlafaxine antinociception.
			Metergoline; s.c.	Hot-plate	No significant changes	To examine the role of serotonergic receptor blockade in venlafaxine antinociception.
Schreiber et al., 2002 [21]	Male ICR mice	30 mg/kg; i.p.	$\mathrm{Yohimbine}\;(\alpha_{2}$ adrenergic antagonist) *	Hot-plate	Antinociception inhibited	To determine whether α_2_-adrenergic receptor blockade modifies venlafaxine antinociception.
			Metergoline (serotoninergic antagonist) *	Hot-plate	No inhibition of antinociceptive effect of venlafaxine	To examine serotonergic receptor involvement in venlafaxine antinociception
			$\mathrm{Phentolamine}\;(\alpha_{1}$α_2_-adrenergic antagonist) *	Hot-plate	No inhibition of antinociceptive effect of venlafaxine	$\mathrm{To}\;\mathrm{determine}\;\mathrm{whether}\;\alpha_{1}$α_2_-adrenergic receptor blockade modifies venlafaxine antinociception.
		2.5 mg/kg; i.p.	$\mathrm{Clonidine}\;(\alpha_{2}$ adrenergic agonist) *	Hot-plate	5-fold ED_50_ reduction 0.5 → 0.1 mg/kg	To evaluate venlafaxine effects on clonidine dose–response antinociception.
			Serotonin *	Hot-plate	No significant changes.	To examine the role of serotonergic receptor involvement in venlafaxine antinociception.
Bonnefont et al., 2005 [32]	Sprague–Dawley rats	2.5 mg/kg; s.c.	WAY-100635 (5-HT_1_A antagonist); i.t.	Formalin test	Partial reversal of antinociception	To investigate the role of spinal 5-HT_1_A receptors in venlafaxine antinociception.
Berrocoso and Mico, 2009 [23]	CD1 mice	40 mg/kg; i.p.	WAY-100635 (5-HT_1_A antagonist); s.c.	Hot-plate	Increased latency of the nociceptive response to thermal stimulus	To evaluate effects of 5-HT_1_A receptor antagonism on venlafaxine antinociception.
		80 mg/kg; i.p.	8-OH-DPAT (5-HT_1_A agonist); s.c.	Hot-plate	No increase in antinociceptive effect	To examine effects of 5-HT_1_A receptor activation on venlafaxine antinociception.
Gultekin & Ahmedov, 2006 [22]	Wistar rats	25 mg/kg; i.p.	L-NOARG; i.p.	Hot-plate	No change in antinociceptive effect	To assess involvement of nitric oxide synthase inhibition in venlafaxine antinociception.
Kothari et al., 2013 [26]	Albino mice	7.5 mg/kg; p.o.	Aegle marmelos; p.o.	Writhing	Significant inhibition of writhes	To evaluate combined effects of venlafaxine and plant-derived extract in visceral nociception.
				Tail-flick	Significant increased latency of the nociceptive response to thermal stimulus	To evaluate combined effects of venlafaxine and plant-derived extract in spinal nociception.
Schreiber et al., 2015 [33]	Male ICR mice	2.5 mg/kg; i.p.	Methylphenidate; i.p.	Hot-plate	Increased latency of the nociceptive response to thermal stimulus	To evaluate interaction between venlafaxine and dopamine–norepinephrine reuptake inhibition.
Mansouri et al., 2015 [28]	Swiss mice	ED_50_ dosing; i.p.	Ellagic acid; i.p.	Writhing	Synergistic antinociceptive interaction	To determine whether an antioxidant compound modifies venlafaxine antinociceptive potency.
Soleimanii et al., 2024 [29]	NMRI mice	20 mg/kg; i.p.	Ca-channel blockers; i.p., and morphine; s.c.	Hot-plate	Enhanced morphine analgesia	To evaluate effects of venlafaxine and calcium channel blockers on the acute analgesic effects of morphine

i.p., intraperitoneal; s.c., subcutaneous; p.o., oral; i.t., intrathecal; ED_50_, median effective dose. Thermal latency: time from stimulus onset to the thermal nociceptive response (e.g., paw licking, jump or tail-flick latency). Writhing inhibition: reduction in abdominal constriction frequency relative to control. *, route of administration is not mentioned in the study.

## Data Availability

No new data were created or analyzed in this study. Data sharing is not applicable to this article.

## Abbreviations

The following abbreviations are used in this manuscript:

5-HT	Serotonin (5-hydroxytryptamine)
5-HT1A	Serotonin receptor subtype 1A
8-OH-DPAT	8-Hydroxy-2-(di-n-propylamino) tetralin (5-HT1A receptor agonist)
α2	Alpha-2 adrenergic receptor
cGMP	Cyclic guanosine monophosphate
CNS	Central nervous system
CYP2D6	Cytochrome P450 2D6
DPDPE	[D-Pen^2^,D-Pen^5^]enkephalin (δ-opioid receptor agonist)
DPMS	Descending pain modulatory system
ED_50_	Median effective dose
i.c.v.	Intracerebroventricular
i.p.	Intraperitoneal
i.t.	Intrathecal
IJMS	International Journal of Molecular Sciences
MPE	Maximal possible effect
NE	Norepinephrine (noradrenaline)
NET	Norepinephrine transporter
NO	Nitric oxide
NO–cGMP	Nitric oxide–cyclic guanosine monophosphate signaling pathway
ODV	O-desmethylvenlafaxine
PAG	Periaqueductal gray
PD	Pharmacodynamics
PK	Pharmacokinetics
PK/PD	Pharmacokinetic–pharmacodynamic
RVM	Rostroventromedial medulla
SERT	Serotonin transporter
SNRI	Serotonin–norepinephrine reuptake inhibitor
U50488H	κ-opioid receptor agonist (U-50,488H)
WAY-100635	Selective 5-HT1A receptor antagonist
